# *CPR63* promotes pyrethroid resistance by increasing cuticle thickness in *Culex pipiens pallens*

**DOI:** 10.1186/s13071-022-05175-0

**Published:** 2022-02-14

**Authors:** Yang Xu, Jingwei Xu, Yang Zhou, Xixi Li, Yufen Meng, Lei Ma, Dan Zhou, Bo Shen, Yan Sun, Changliang Zhu

**Affiliations:** 1 Department of Pathogen Biology, Nanjing Medical University, Nanjing, China; 2 School of Medicine & Holistic Integrative Medicine, Nanjing University of Chinese Medicine, Nanjing, China

**Keywords:** CPRs, Deltamethrin, Mosquito, *Culex pipiens pallens*, Cuticular resistance, Cuticle thickening

## Abstract

The cuticle protein (CP) encoded by *CPR63* plays a role in deltamethrin resistance in *Culex pipiens pallens*. Herein, we investigated the distribution of *CPR63* transcripts in this organism and observed high expression levels in legs and wings. Furthermore, expression of *CPR63* in the legs of deltamethrin-resistant (DR) strains was 2.17-fold higher than in deltamethrin-susceptible (DS) strains. Cuticle analysis of small interfering RNA (siRNA) groups by scanning electron microscopy (SEM) revealed a significantly thinner cuticle of the tarsi in the siCPR63 group than in the siNC (negative control siRNA) group. Transmission electron microscopy (TEM) revealed that the exocuticle and endocuticle thickness of the tarsi were significantly thinner, which contributes the thinner procuticle of tarsi in the siCPR63 group than in the siNC group. Our results suggested that CPR63 might contribute to the resistance phenotype by thickening the cuticle and thereby possibly increasing the tolerance of mosquitoes to deltamethrin.

**Supplementary Information:**

The online version contains supplementary material available at 10.1186/s13071-022-05175-0.

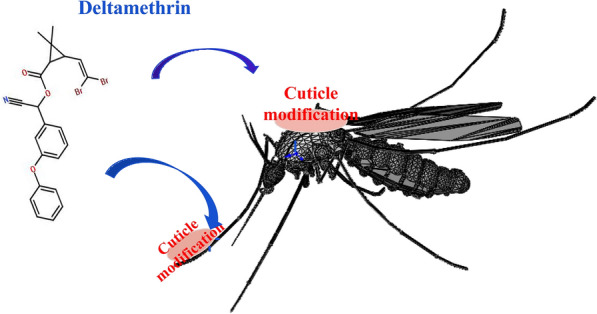

## Introduction

Targeting insect vectors has proven to be the most effective means for preventing the spread of mosquito-borne diseases [1, 2]. Chemical insecticides are the most important component in this effort. However, the spread of insecticide resistance seriously threatens the success and sustainability of control interventions [3]. According to the latest World malaria report, 73 countries reported mosquito resistance to at least one of the four commonly used insecticide classes during 2010−2019, and 28 countries reported mosquito resistance to all major insecticide classes [4]. Therefore, it is critical to develop and apply effective insecticide resistance management strategies.

Research on the mechanism of mosquito vector resistance is of great significance for mosquito vector control. Generally, insecticide resistance in insects is caused by three major mechanisms: (i) reduced sensitivity of the target site, (ii) increased activity and/or abundance of detoxification enzymes and (iii) reduced penetration of insecticides due to altered cuticles [5]. The cuticle is believed to function in insecticide resistance by reducing or slowing insecticide uptake. Various CPs (CPR, CPAPn, CPG, CPF and CPLCG) belonging to different protein families have been identified [6, 7]. Some cuticle proteins (CPs) play major roles in insecticide resistance in mosquitoes. For example, previously, CPLCG3, CPLCG4 and CPLCG5 were involved in a putative cuticle thickening mechanism. CPR124, CPR127, CPR129 and CPR131 were found expressed at higher levels in pyrethroid-resistant compared to susceptible mosquitoes [14, 24, 25, 27]. Most CPs belong to the CPR family and possess characteristic Rebers and Riddiford (R&R) consensus sequences (RR-1 and RR-2) that in an extended form confer chitin-binding properties [8–11]. In a recent study, the location of RR-1 s and RR-2 s was found to be more dependent on the properties of individual proteins than had been reported in previous work [12]. A previous study in our laboratory investigated expression of the CPR63 gene (GenBank: MF095856.1), encoding an RR-2 family member in *Cx. pipiens pallens*, and this was more abundant in deltamethrin-resistant (DR) than deltamethrin-susceptible (DS) strains [13]. Furthermore, the mosquito mortality rate was altered by silencing the *CPR63* gene [13]. However, the detailed resistance mechanism of CPR63 related to the mosquito cuticle remains unknown. In this study, we revealed that CPR63 might participate in pyrethroid resistance by thickening the cuticle and thereby possibly increasing the tolerance of mosquitoes to deltamethrin.

## Materials and methods

### Mosquito strains

In this study, we collected *Cx. pipiens pallens* from Tangkou (Shandong Province, China) as DS strains, and the LC_50_ for deltamethrin for these DS strains was 0.03 mg/l. DR strains were isolated from DS strains with an LC_50_ of 7.5 mg/l by repeatedly selecting 84 generations at the larval stage. Other details were as described in a previous study [14].

### RNA extraction and cDNA synthesis

We collected heads, thorax, abdomen, and all legs and wings of the DS and DR female mosquitoes at 72 h post-eclosion (PE) in three tubes for each tissue (20 mosquitoes per tube). Extraction of mosquito total RNA was performed according to the RNAiso Plus instructions (Takara, Shiga, Japan), and the RNA was then converted to cDNA using the PrimeScriptRT Reagent Kit (TaKaRa, Tokyo, Japan).

### Quantitative real-time PCR (qPCR)

cDNA samples were diluted properly with RNase-free water before use as templates in the quantitative PCR process using SYBR Green (Applied Biosystems, Foster City, CA, USA) according to the manufacturer’s protocol. The reaction volume (20 μl) contained the Power SYBR Green PCR Master Mix, specific forward and reverse primers (Additional file 3: Table S1) and diluted cDNA. The PCR conditions were as follows: 50 °C for 2 min and 95 °C for 10 min, followed by 40 cycles at 95 °C for 15 s and 60 °C for 1 min. For qPCR validation, the melting curve program was run immediately after the qPCR program showed a single-peaked curve. Amplification signals in the no template or primer control samples were high Ct values (Ct > 35). When the primers were used at the first time, qPCR products were sequenced for confirmation. The correlation coefficients of the calibration curves in each test were > 0.99. The relative expression levels were normalised to the internal control *β*-actin by using the 2^−ΔΔCt^ method [15–18]: target gene/*β*-actin = 2^ΔCt^, ΔCt = Ct_β-actin_ − Ct_target gene_. Three technical and biological replicates were performed for qPCR analyses.

### Gene silencing

Mosquitoes for RNA interference (RNAi) experiments were derived from DR and DS female strains microinjected at 12 h PE, with three tubes for each group (10 RNAi mosquitoes per tube). A small interfering RNA (siRNA) targeting *CPR63* (siCPR63) and a siNC (negative control siRNA) were synthesised by GenePharma (Shanghai, China; Additional file 3: Table S1). The siNC does not cause any gene silencing and has no homologous genes in the mosquito gene bank. About 364 ng of siCPR63 and 350 ng of siNC were separately injected into the thorax of female mosquitoes. Other details of the gene silencing method have been described previously [15]. After 3 days, qPCR was performed to determine the interference efficiency of the target gene.

### Scanning electron microscopy (SEM)

To avoid the influence of mosquito size on cuticle thickness, we measured the wing length of all female mosquitoes in the experiment [31]. There were 11 female mosquitoes in each group (siNC, siCPR63), and we selected one right front leg from each female mosquito. All microinjected mosquito legs were washed twice in 70% ethanol to clean them thoroughly. Alcohol was dripped onto tarsomere I of the right front leg at the midpoint, and the leg was cut with a new platinum-coated blade and washed again to remove any debris. Legs were then fixed in 2.5% glutaraldehyde (Sigma, St Louis, MO, USA) for 12 h and incubated for 10 min each in a graded ethanol series (30, 50, 70, 80, 90, 95 and 100%). Legs were dried in an EM CPD300 critical point dryer (Leica, Wetzlar, Germany) using an automated process for 15 exchanges. A K550 X sputter coater (Electron Microscopy Sciences, Hatfield, PA, USA) was used for coating samples. A Quanta 250 FEI scanning electron microscope was employed, and images were recorded at a 3-kV acceleration voltage. The thickness of the cuticle was examined using image J software (http://imagej.net/Welcome). The average cuticle thickness of each leg was calculated by measuring the distance at 23 randomly selected points.

### Transmission electron microscopy (TEM)

Six female mosquitoes were included in each group (siNC, siCPR63), and we selected one right front leg from each female mosquito (i.e. six legs per group). Tarsi were divided into four equal parts, and 2–3 images were captured for each part, resulting in 9–10 images for each leg and 58 images in total. The thickness of the cuticle was examined using image J software. According to the obtained pictures, we also counted the number of pores in tarsi. The other detailed steps of the TEM experiment have been described in previous studies [14, 19].

### Statistical analysis

Experimental data between two groups were analysed using Student’s *t*-test. The expression levels of *CPR63* in different tissues were calculated using an ANOVA test. All data are presented as the mean ± standard deviation (SD), and *p* < 0.05 was considered statistically significant. All experiments were performed using at least three independent cohorts.

## Results

### CPR63 transcripts are abundant in DR mosquito legs

**Fig. 1:**
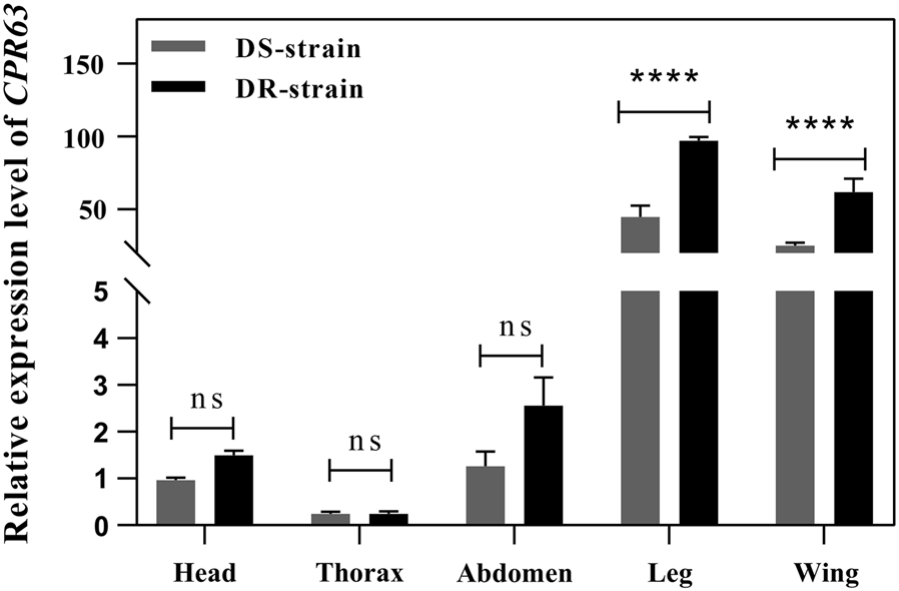
Expression profiles of *CPR63* in different mosquito tissues. Constitutive expression of *CPR63* in DS and DR strains; mRNA expression levels were measured in the head, thorax, abdomen, legs and wings in DS and DR strain mosquitos. Head of DS strain was ascribed an arbitrary value of 1. Results are presented as the mean ± standard deviation (SD) of three biological replicates. ****p ≤ 0.0001; ns, not signifcant, *p* > 0.05

To explore the function of *CPR63*, we examined localisation of *CPR63* expression in multiple tissues at 72 h PE by qPCR in DS and DR strains, including the heads, thorax, abdomen, legs and wings. The results showed that *CPR63* was highly expressed in legs. Expression of *CPR63* in the legs of DR strains was 2.17-fold higher (ANOVA, *p* < 0.0001) than those in DS strains (Fig. 1). The result showed that *CPR63* was highly expressed in legs and indicated that *CPR63* might play an important role in leg resistance.

### Expression of CPR63 following gene silencing

**Fig. 2:**
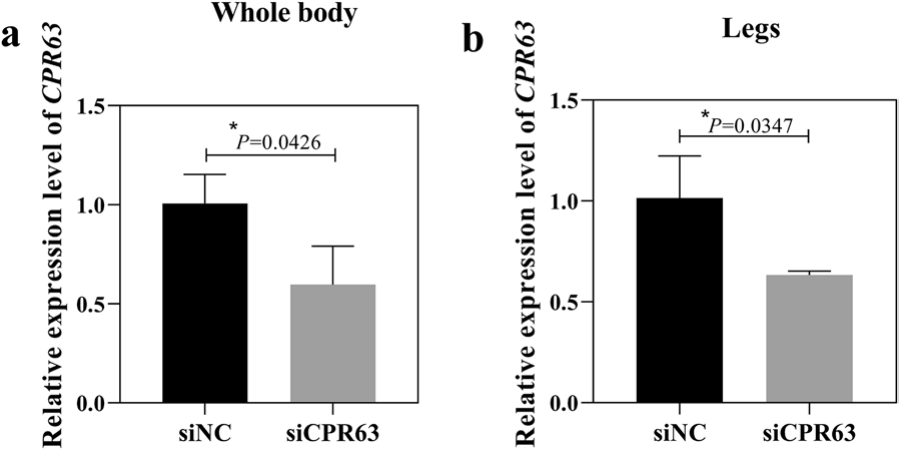
Relative expression levels of *CPR63* after RNAi silencing. Levels of *CPR63* expression in whole mosquito bodies (**a**) and legs (**b**) after silencing of *CPR63* were measured by qPCR. Results are shown as the mean ± SD of three biological replicates. *p ≤ 0.05; **p ≤ 0.01

Expression of *CPR63* was evaluated by qPCR in DR strains at 12 h PE after injection of siRNA targeting the *CPR63* gene. Expression of *CPR63* was significantly decreased by 40.9% (*t*-test, *t*_(4)_ = 2.935, *p* = 0.0426) in the whole bodies of mosquitoes and by 37.6% (*t*-test, *t*_(4)_ = 3.146, *p* = 0.0347) in the legs at 72 h PE after siCPR63 injection compared to injection with siNC (Fig. 2a, b). We also found that interference efficiency of *CPR6*3 in DS strains is not statistically significant compared with siNC group of DS strains, which could be used as a control (Additional file 2: Figure S2).

### SEM analysis of cuticle thickness

**Fig. 3:**
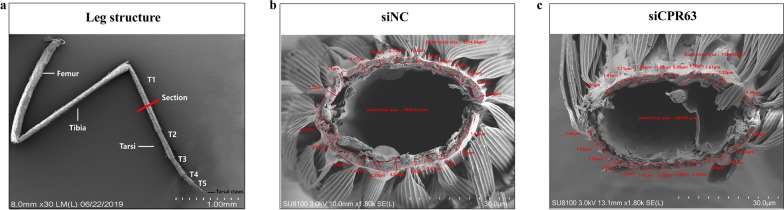
SEM analysis of the effects of siNC and siCPR63. **a** Illustration of the position of sectioning on the *Cx. pipiens pallens* tarsomere 1 (t1–t5 = five tarsal segments) [20]. The red line indicates in which leg part the sections were taken. The SEM images show a front view of a sectioned leg for the siNC group (**b**) and the siCPR63 group (**c**)

**Fig. 4:**
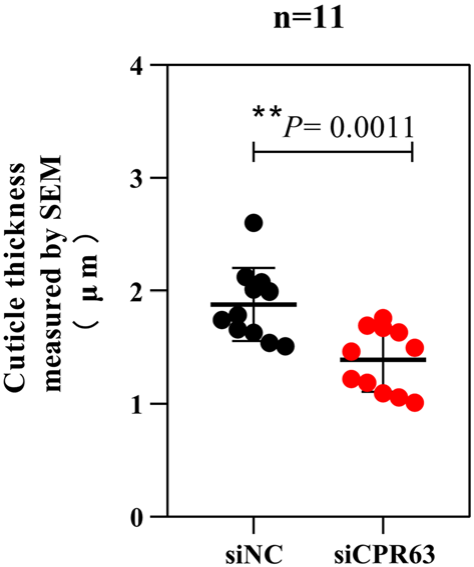
SEM analysis of cuticle thickness. Measurements were made at 23 different points per individual, allowing for the calculation of mean cuticle thickness. Results are shown as the mean ± SD; *n* = the number of measurements carried out on each group of 11 mosquitoes. **p = 0.0011

To probe the changes in the overall cuticle structure, the region of the tarsus segment was analysed by SEM. The mosquito leg is composed of the femur, tibia and tarsus, the tarsus is divided into T1, T2, T3, T4 and T5, and the cross section was at T1 (Fig. 3a) [19, 20]. The same number of specimens was assessed in siCPR63 and siNC groups (*n* = 11). Mosquito leg tarsi were assessed by comparing the size of the mosquito wing and measuring the area of the inner and outer circles of the mosquito leg cuticle. The results showed that the tarsi were a similar size in the two groups, but they were significantly thinner in the siCPR63 than in the siNC group (Fig. 3b, c). The cuticle thickness was measured at no fewer than 23 random points to obtain the average cuticle thickness of the tarsus. The measurement results showed that the mean cuticle thickness of the siCPR63 group (1.354 ± 0.23 μm) was thinner than that of the siNC group (2.006 ± 0.73 μm; Fig. 4; Table 1; *t*-test, *t*_(44)_ = 3.981, *p* = 0.0011).

**Table 1:** Average cuticle thickness of each component

Cuticle	Procuticle thickness by SEM (μm)	Procuticle thickness by TEM (μm)	Edocuticle thickness by TEM (μm)	Exocuticle thickness by TEM (μm)
Group				
siNC	2.006 ± 0.73	3.239 ± 0.74	1.489 ± 0.30	1.609 ± 0.22
siCPR63	1.354 ± 0.23	2.219 ± 0.86	0.924 ± 0.48	1.337 ± 0.36

### Ultrastructure analysis of tarsi segment cuticles in siCPR63 and siNC mosquitoes by TEM

**Fig. 5:**
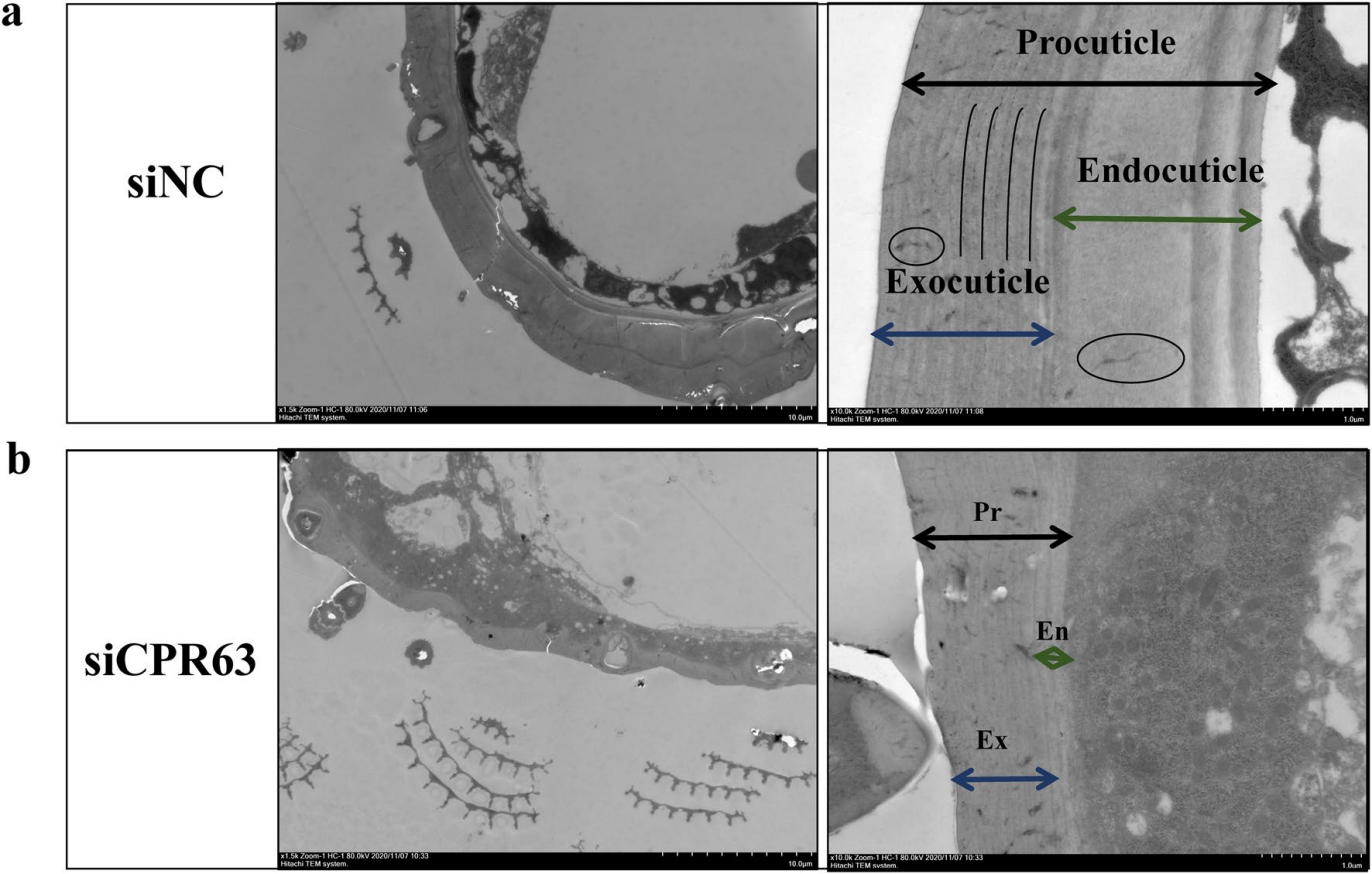
TEM analysis of the effects of siNC and siCPR63. SEM images show a front view of a sectioned leg for siNC (**a**) and siCPR63 (**b**) groups. The curve represents the chitin parallel laminae, and the circle represents the pores

**Fig. 6:**
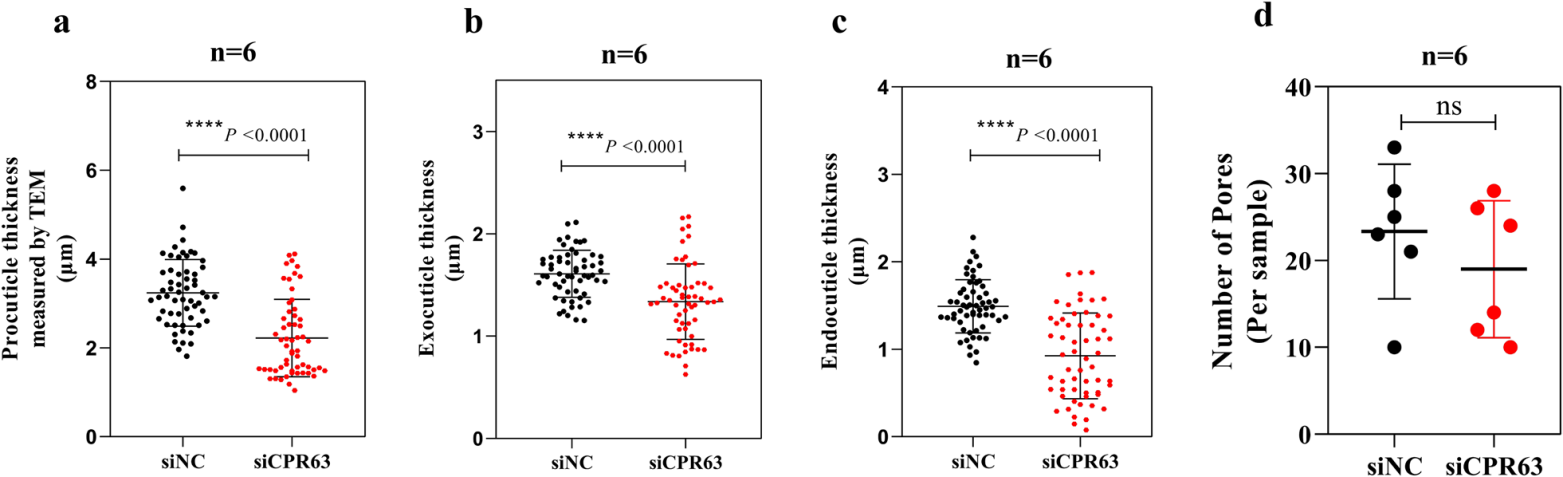
TEM analysis of cuticle thickness. Measurements were performed at 58 points per individual, allowing for the calculation of mean cuticle thickness. Results are shown as the mean ± SD; *n* = the number of measurements carried out on each batch of six mosquitoes. ****p ≤ 0.0001; not signifcant, *p* > 0.05

To explore differences in the cuticle ultrastructure of mosquitoes, the region of the tarsus segment was analysed by TEM. Additional file 1: Figure S1 shows a schematic diagram of how images were captured. The leg cuticle is mainly composed of the procuticle, which is divided into the exocuticle and the endocuticle (Fig. 5). The results showed that the procuticle thickness of the siCPR63 group (2.219 ± 0.86 μm) was thinner than that of the siNC group (3.23 ± 0.74 μm; Fig. 6a; Table 1; *t*_(114)_ = 6.756, *p* < 0.0001). The exocuticle and endocuticle thickness of the leg tarsus in the siCPR63 was thinner than in the siNC group (Fig. 6b, c; Table 1). Compared with the siNC group, the chitinous parallel laminae, number and size of pores in the siCPR63 group were comparable to the siNC group (Figs. 5a, b; 6d).

## Discussion

Cuticle proteins play an important role in insect cuticle resistance, by thickening the cuticle to prevent the penetration of insecticides and changing the density, thickness and insect morphological development of the cuticle. There is increasing evidence that alteration of the cuticle plays a role in insecticide resistance, based on analysis of CP transcripts and measurement of cuticle thickness [19, 21–25]. For example, *CYP4G16*, *CPLCG3*, *CPLCG5* and *CPLC8* have been implicated in insecticide resistance by contributing to a thicker cuticle and thereby slowing penetration of insecticides [14, 21, 24–26]. Three CPR genes (*CPR124*, *CPR129* and *CPR127*) were found to be constitutively overexpressed in resistant *Anopheles gambiae* [25]. In another study, 31 cuticle proteins were differentially regulated in the leg proteome, of which 29 including CPR106, CPR126, CPR121 and CPR151 were overexpressed, and only 2 were downregulated [22]. Furthermore, > 65% of differentially expressed CPs belonged to the CPR family. Strong overexpression of cuticle protein CPR131 was also reported in multi-insecticide-resistant *A. gambiae* [27], and *CPR63*, *CPR47*, *CPR48*, *CPR45* and *CPR44* are highly expressed in DR strains of *Cx. pipiens pallens* [13]*.*

Overexpression of CPRs in resistant mosquitoes has been widely reported, but their cuticle resistance mechanisms remain poorly understood. Our previous study found that silencing the *CPR63* gene made mosquitoes more susceptible to deltamethrin, suggesting that *CPR63* participates in pyrethroid resistance [13]. In the present study, our results led us to speculate about the resistance mechanism by which *CPR63* might contribute to the resistance phenotype; *CPR63* is involved in thickening of the cuticle, and thereby possibly increasing the tolerance of mosquitoes to deltamethrin.

Insect CPs are diverse and expressed in the head, thorax and abdomen. Some CPs are also highly expressed in insect legs. For example, CPLCG5 is highly expressed in the legs of *Cx. pipiens pallens* [14], and members of the CPCFC CP family in *A. gambiae* are mainly distributed in legs [28]. Noh et al. found that cuticle protein TcCPR4 in *Tribolium castaneum* was mainly enriched in the legs and participated in the formation of pore canals in the rigid cuticle [8]. *CPF3*, *CPLCG3*, *CPLCG4* and *CPLCG5* mRNA transcripts were mainly located in appendages (legs and wings) [14, 24]. Similarly, in the present study, *CPR63* mRNAs were mainly located in mosquito legs and wings. Since these appendages are associated with motion, *CPR63* might be related to flight. Additionally, *CPR63* was expressed more highly in the legs of DR strains, indicating that it might help mosquitoes avoid areas treated with insecticides, but this hypothesis requires further exploration.

Different CPs play different roles in cuticular resistance. Huang et al. reported that CPLCG5 acts as a major CP and is highly expressed in the legs in *Cx. pipiens pallens* [14]. Our current results showed that expression of *CPR63* was increased in insecticide-resistant *Cx. pipiens pallens* and also highly expressed in the legs. In addition, silencing of *CPLCG5* resulted in larger pore canals, indistinct chitinous parallel laminae and thinner endocuticle in the leg structures. Specifically, silencing of *CPR63* resulted in thinner endocuticle and exocuticle, but the chitinous parallel laminae and number and size of pores are not significantly altered, indicating that different CPs perform distinct functions to contribute to cuticular resistance, and *CPR63* participates in cuticular resistance mainly by increasing the cuticle thickness.

Early studies suggest that RR-1 and RR-2 proteins are present in different regions within the cuticle itself; RR-2 proteins contribute to exocuticle, and RR-1 proteins are found in the endocuticle [29, 30]. However, a more recent study showed that the location of RR-1 s and RR-2 s depends more on the properties of individual proteins [12]. Our current study showed that silencing *CPR63* led to thinner endocuticle and exocuticle. We therefore speculate that CPR63 may be distributed in both the endocuticle and exocuticle, but this hypothesis needs further verification. In addition, our previous study found that another cuticle protein, CPR47, is also related to insecticide resistance. There may be an interaction between cuticle proteins, but how this affects resistance and whether it is related to CPR63 remain unknown. The work needs further study.

In summary, our results revealed that CPR63 might participate in pyrethroid resistance by thickening the cuticle and thereby possibly increasing the tolerance of mosquitoes to deltamethrin. This is the first report linking CPRs to insecticide resistance in mosquito legs.

## Supplementary Information

**Additional file 1: Figure S1.** Schematic diagram showing image capture.

**Additional file 2: Figure S2.** Relative expression levels of *CPR63* after RNAi silencing in DS strains.

**Additional file 3: Table S1.** Primers used for qPCR analysis and siRNA synthesis of *CPR63*.

## Data Availability

All data are fully available without restriction.

## Abbreviations

DR: Deltamethrin-resistant
DS: Deltamethrin-susceptible
PE: Post-eclosion
CPs: Cuticle proteins
R&R: Rebers and Riddiford
qPCR: Quantitative real-time PCR
siRNA: Small interfering RNA
siCPR63: Small interfering RNA for silencing the *CPR63* gene
NC: Negative control
siNC: Negative control siRNA
SEM: Scanning electron microscopy
TEM: Transmission electron microscopy
