# Supplementary figures and images for: CPR63 promotes pyrethroid resistance by increasing cuticle thickness in Culex pipiens pallens
Source: Parasit Vectors. 2022 Feb 14;15:54. doi: 10.1186/s13071-022-05175-0 (PMC8842966; doi:10.1186/s13071-022-05175-0)

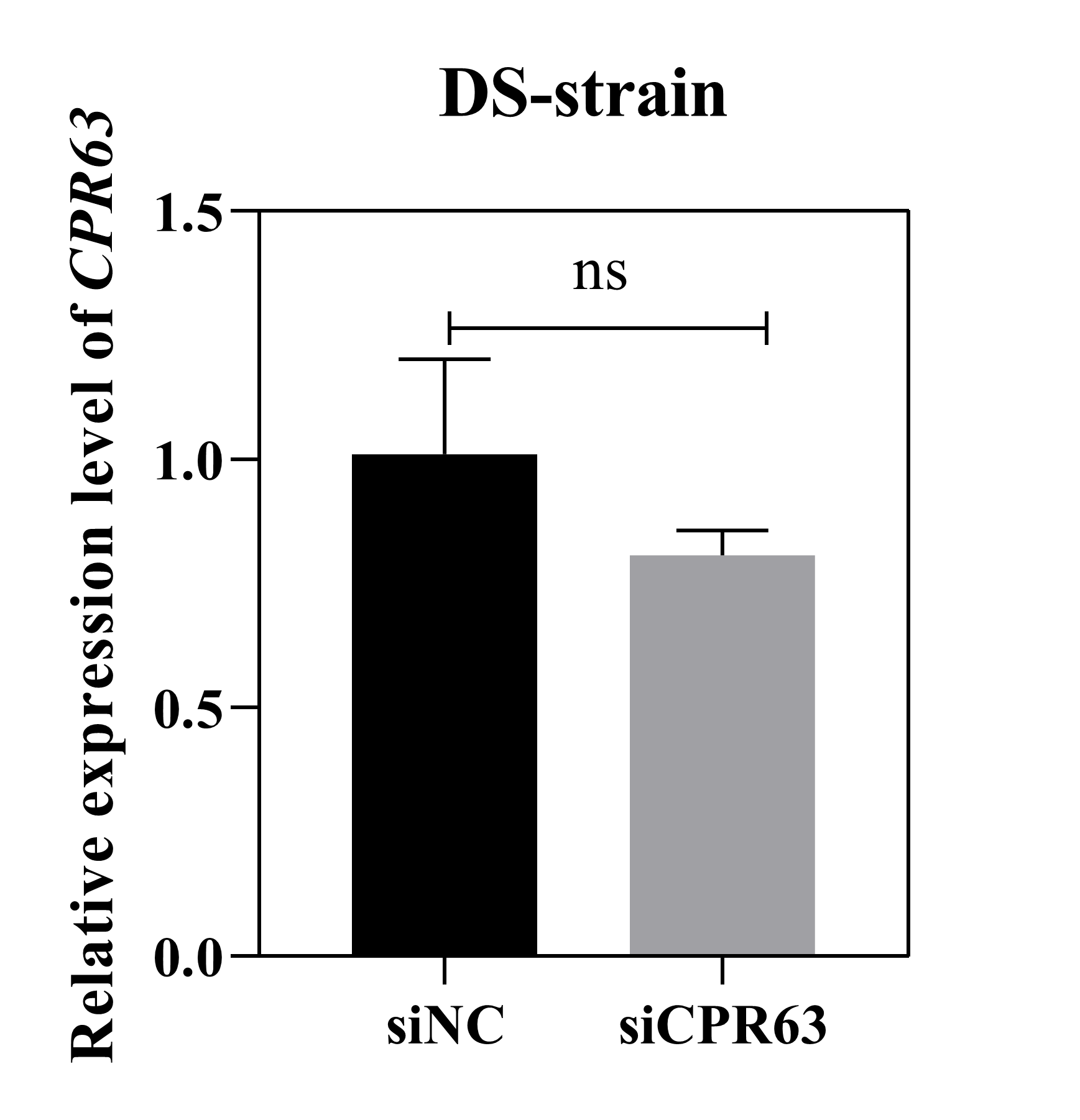

Supplement: Supplementary file 2 — Additional file 2: Figure S2. Relative expression levels of CPR63 after RNAi silencing in DS strains. [file 13071_2022_5175_MOESM2_ESM.tif]
