# Supplementary material for: CPR63 promotes pyrethroid resistance by increasing cuticle thickness in Culex pipiens pallens
Source: Parasit Vectors. 2022 Feb 14;15:54. doi: 10.1186/s13071-022-05175-0 (PMC8842966; doi:10.1186/s13071-022-05175-0)
Supplement: Supplementary file 3 — Additional file 3: Table S1. Primers used for qPCR analysis and siRNA synthesis of CPR63. [file 13071_2022_5175_MOESM3_ESM.docx]

**Table S1**. **Primers used for qPCR analysis and siRNA synthesis of *CPR63***

| Application of primers | Primer name | Primer sequence (5’ to 3’) |
| --- | --- | --- |
| qRT-PCR | CPR63-F | GCATTGGTTGCCTGTTTG |
|  | CPR63-R | TGCTCCCACTGGCTCTTGT |
|  | β-actin-F | AGCGTGAACTGACGGCTCTTG |
|  | β-actin-R | ACTCGTCGTACTCCTGCTTGG |
| siRNA synthesis | siCPR63-F | GAACAUUGAUCAGCACCAUTT |
|  | siCPR63-R | AUGGUGCUGAUCAAUGUUCTT |
|  | siNC-F | UUCUCCGAACGUGUCACGUTT |
|  | siNC-R | ACGUGACACGUUCGGAGAATT |

F=Forward; R=Reverse
